# Integrated omics-driven discovery reveals synphilin-1-mediated anoikis resistance through p53 down-regulation

**DOI:** 10.1042/BSR20260173

**Published:** 2026-09-18

**Authors:** Seok Gi Kim, Ye Eun Sim, Abdurazak Aman Ketebo, Ji Su Hwang, Nimisha Pradeep George, Yong Eun Jang, Minjun Kwon, Sungsu Park, Jung-Soon Mo, Gwang Lee

**Affiliations:** 1 Department of Physiology, Ajou University School of Medicine, 164 World Cup-ro, Suwon 16499, Republic of Korea; 2 Department of Biomedical Sciences, BK21 R&E Initiative for Advanced Precision Medicine, Graduate School of Ajou University, 164 World cup-ro, Suwon 16499, Republic of Korea; 3 Department of Mechanical Engineering, Sungkyunkwan University, 2066 Seobu-ro, Suwon 16419, Republic of Korea; 4 Department of Molecular Science and Technology, Ajou University, 206 World cup-ro, Suwon 16499, Republic of Korea; 5 Institute of Quantum Biophysics (IQB), Sungkyunkwan University, 2066 Seobu-ro, Suwon 16419, Republic of Korea; 6 Department of MetaBioHealth, Sungkyunkwan University, 2066 Seobu-ro, Suwon 16419, Republic of Korea; 7 Institute of Medical Science, Ajou University School of Medicine, 164 World cup-ro, Suwon 16499, Republic of Korea

**Keywords:** Anoikis, Integrated omics, p53, Parkinson’s disease, Synphilin-1

## Abstract

Synphilin-1 is a protein that interacts with α-synuclein and has been implicated in Parkinson’s disease (PD). However, the cellular and molecular mechanisms underlying the effect of synphilin-1 in PD remain poorly understood. The present study aimed to elucidate the molecular function of synphilin-1 using integrated transcriptomic and proteomic *in silico* analyses, followed by *in vitro* validation. Synphilin-1 overexpression enhanced cell viability and attenuated pathways associated with cell death. Among the identified regulatory molecules, p53 emerged as a key mediator linking synphilin-1 to suppression of anoikis. Notably, p53 expression demonstrated predominant nuclear localisation in midbrain tissues of individuals with PD. These findings suggest that synphilin-1 promotes cell survival by suppressing p53-mediated anoikis. This regulatory relationship could be crucial for understanding neuroprotective or pathological mechanisms in PD.

## Introduction

Synphilin-1 is an α-synuclein-interacting cytoplasmic protein that is a component of Lewy bodies and has been implicated in Parkinson’s disease (PD) pathology [1,2]. Synphilin-1 was found to exert neuroprotective effects by mitigating neurotoxic responses induced by PD-mimetic neurotoxins in experimental PD models [3–7]. However, the precise cellular and molecular mechanisms underlying the protective effect of synphilin-1 in neurons against PD-associated pathogenic stress remain unclear.

Anoikis is a specialised form of apoptosis that occurs when anchorage-dependent cells detach from the extracellular matrix (ECM) or adhere to inappropriate substrates [8]. Anoikis was first described in epithelial and endothelial cells, and it plays a critical role in maintaining tissue development and homeostasis by eliminating detached cells and preventing aberrant re-adhesion [9]. Although anoikis failure has emerged as a hallmark of cancer cells [10–14], its relevance extends to other diseases. Anoikis-related gene signatures are associated with ophthalmic disorders, inflammatory bowel disease, and PD [15–17]. In the nervous system, anoikis-like mechanisms have been implicated in hippocampal neuronal death [18], whereas disturbances in ECM–neuron and neuron–astrocyte interactions impair neuronal metabolism, thereby driving neurodegeneration [19]. These findings raise the possibility of an unrecognised role for anoikis in neuronal vulnerability and PD pathogenesis.

## Hypothesis

We hypothesised that synphilin-1 overexpression promotes resistance to anoikis through the down-regulation of p53, thereby enhancing cellular survival in the context of PD. We further proposed that altered nuclear localisation of p53 in the human midbrain reflects dysregulation of this pathway and supports its pathological relevance in PD. This regulatory interaction may represent a key mechanism underlying the neuroprotective effects of synphilin-1 in PD.

## Methods

### Cell culture and transfection

Human embryonic kidney 293 (HEK293) and human neuroblastoma SH-SY5Y cell lines were purchased from the American Type Culture Collection. Although originally derived from embryonic kidney, HEK293 cells share several molecular and morphological features with neuronal lineage cell models such as NTERA-2 cells and express neurofilament (NF) subunits, including NF-M, NF-L, and NF-H [20]. Additionally, Synph-293 cells, stably overexpressing Flag-synphilin-1 and derived from HEK293 cells, have been used to evaluate the formation of cytoplasmic inclusions that are characteristic of neurodegenerative disorders [21], owing to their high propensity to develop inclusion-like structures under stressful conditions. HEK293, Synph-293, and SH-SY5Y cells were cultured in the appropriate media [Dulbecco’s modified Eagle medium (DMEM) for HEK293 and Synph-293 cells; DMEM/F12 for SH-SY5Y cells], supplemented with 10% foetal bovine serum (FBS; Corning, NY, U.S.A.), penicillin (100 units/ml), and streptomycin (100 μg/ml) (Thermo Fisher Scientific, Waltham, MA, U.S.A.). The cells were incubated at 37°C in a humidified incubator under 5% CO_2_. The HEK293 and SH-SY5Y cells were transfected with an empty vector or a Flag-synphilin-1 vector using Lipofectamine 3000 (Invitrogen, Waltham, MA, U.S.A.), according to the manufacturer’s instructions. Synphilin-1 knockdown was performed by transfecting the scrambled control siRNA or synphilin-1 siRNA (Dharmacon, Lafayette, CO, U.S.A.) into cells using Lipofectamine RNAiMAX (Invitrogen), according to the manufacturer’s instructions.

### RNA sequencing

RNA sequencing was performed using two independent biological replicates per experimental group, each prepared as an individual library and sequenced once [22]. Briefly, total RNA (1 μg) was used to generate libraries using the TruSeq Stranded mRNA Sample Prep Kit (Illumina, San Diego, CA, U.S.A.). Library quality and quantity were assessed using an Agilent 2100 BioAnalyzer (Agilent Technologies, Palo Alto, CA, U.S.A.) and the KAPA library quantification kit (Kapa Biosystems, Wilmington, MA, U.S.A.). Sequencing was performed on an Illumina NovaSeq 6000 platform (paired-end, 2 × 150 bp). Differentially expressed genes were selected based on the significance criteria *P* < 0.05 and |fold change| > 1.2. Transcriptome sequencing and processed data have been deposited in the Gene Expression Omnibus (GEO) database under the accession number GSE255201.

### Proteomic analysis

Tandem mass tag (TMT)-based quantitative proteomic analysis was performed using three independent biological replicates per group, each processed and analysed once [22]. Briefly, HEK293 and Synph-293 cells were lysed in RIPA buffer (Thermo Fisher Scientific), and the samples were denatured, reduced, alkylated, and digested with trypsin. The peptides were labelled with a TMT 6-plex (Thermo Fisher Scientific) and fractionated using high-pH reversed-phase liquid chromatography (Nexera XR; Shimadzu, Kyoto, Japan) on a C_18_ column. TMT-labelled samples were analysed using an Orbitrap Fusion Lumos mass spectrometer and a nanoelectrospray source with an Easy nLC 1200 (Thermo Fisher Scientific). TMT-based quantitative analysis was performed using ProLucid [23] and DTASelect [24] for protein identification and Census within the IP2 pipeline (Integrated Proteomics, U.S.A.) for quantification. MS/MS spectra were searched against the UniProt database (January 2018, reviewed proteins) using ProLucid. Reversed protein sequences were appended to the database to estimate the false discovery rate (FDR). The data were filtered using DTASelect to generate the protein list, requiring at least two peptide assignments and an FDR of less than 0.01. Differentially expressed proteins were selected based on the significance criteria *P* < 0.05 and |fold change| > 1.2. Proteomic data have been deposited in the MassIVE database under the accession number MSV000089926.

### Pillar array fabrication and traction force measurement

Polydimethylsiloxane (PDMS) pillar arrays were fabricated using a previously described platform [25,26]. A silicon wafer mould containing a linear array of holes was fabricated using photolithography and reactive-ion etching. The PDMS mixture was prepared by mixing the elastomer base with a curing agent at a 10:1 ratio (Sylgard 184, Dow Corning, Midland, MI, U.S.A.), followed by degassing for 15 min. Then, the mixture was spin-coated on the mould at 1000 rpm for 1 min, degassed, and cured at 80°C for 3 h. The cured PDMS was detached from the mould in isopropanol, which yielded an array of pillars with the following specifications: diameter, 900 nm; height, 1 μm; and centre-to-centre distance, 1.8 μm. The Young’s modulus of the cured PDMS was 2 MPa, and the bending stiffness of the pillar was 24.2 nN/μm. The pillar arrays were coated with fibronectin (10 μg/ml).

For traction force measurement, images of cells on a pillar array were acquired at 37°C and 5% CO_2_ in a live cell chamber at 1 Hz using a fluorescence microscope (Deltavision, GE Healthcare, Chicago, IL, U.S.A.) equipped with a CoolSNAP HQ^2^ camera (Photometrics, Tucson, AZ, U.S.A.). The pillar positions were tracked using the PillarTracker plugin (v1.1.3, Mechanobiology Institute, Singapore) in ImageJ software (version 1.53t, National Institutes of Health, Bethesda, MD, U.S.A.), which reconstructed the pillar grid and automatically detected pillar displacements. The traction force on each pillar was calculated by multiplying pillar displacement by bending stiffness.

### Flow cytometry

To induce anoikis, the cells were cultured for 3 days in ultra-low-attachment six-well plates (Merck, Darmstadt, Germany). Then, the cells were collected and stained with propidium iodide and fluorescein isothiocyanate (FITC)-Annexin V using the FITC Annexin V Apoptosis Detection Kit (BD Biosciences, San Jose, CA, U.S.A.), according to the manufacturer’s protocol. The flow cytometric data were acquired and analysed using BD FACSCanto with FACSDiva software (BD Biosciences).

### Western blotting

Cells were lysed in RIPA buffer (Thermo Fisher Scientific) supplemented with Protease Inhibitor Cocktail Solution (GenDEPOT, Katy, TX, U.S.A.) for 30 min at 4°C. The protein concentration of the cell lysates was measured using a BCA Protein Assay Kit (Thermo Fisher Scientific). Equal amounts of protein (7 μg/lane) were mixed with a Lane Marker Reducing Sample Buffer (Thermo Fisher Scientific), heated at 70°C for 15 min, cooled down at 4°C for 5 min, and subjected to sodium dodecyl sulphate–polyacrylamide gel electrophoresis. Separated proteins were transferred onto a polyvinylidene difluoride membrane and blocked with 3% skim milk for 1 h at 25°C. The following primary antibodies were used for western blotting: anti-synphilin-1 (1:1000) (sc-365741; Santa Cruz Biotechnology, Dallas, TX, U.S.A.), anti-p53 (1:1000) (sc-126; Santa Cruz Biotechnology), and anti-β-actin (1:2000) (sc-47778; Santa Cruz Biotechnology). For the secondary antibody, a horseradish peroxidase (HRP)-conjugated anti-mouse IgG (sc-516102; Santa Cruz Biotechnology) was used, and the chemiluminescent signals were detected using a SuperSignal™ West Pico PLUS Chemiluminescent Substrate (34580; Thermo Fisher Scientific) and the iBright™ CL1500 Imaging System (Thermo Fisher Scientific).

### Immunofluorescence staining

Cells were seeded onto coverslips in culture plates, fixed with 2% formaldehyde for 15 min at 25°C, and permeabilised with 0.2% Triton X-100 for 5 min. For F-actin staining, the cells were incubated with rhodamine phalloidin (1:1000) (Sigma–Aldrich, St. Louis, MO, U.S.A.) or Alexa Fluor 488 phalloidin (1:250) (Invitrogen) in phosphate-buffered saline (PBS) at 25°C for 30 min. For immunofluorescence staining, the cells were blocked with 10% FBS in PBS for 30 min and incubated at 4°C for 16 h with the primary synphilin-1 antibody (1:100) (sc-365741; Santa Cruz Biotechnology) diluted in blocking buffer. Next, the cells were incubated at 25°C for 90 min with the following secondary antibodies: Alexa Fluor 488 conjugated anti-mouse IgG (Invitrogen) or Alexa Fluor 594 conjugated anti-rabbit IgG (Invitrogen). Then, they were counterstained with 4′,6-diamidino-2-phenylindole for 2 min. The coverslips were mounted using Gel/Mount (Biomeda, San Jose, CA, U.S.A.) and imaged using a Nikon A1R microscope (Nikon, Tokyo, Japan) at the Three-Dimensional Immune System Imaging Core Facility of Ajou University (Suwon, Republic of Korea).

### Tissues and ethical approval

Postmortem human brain specimens from patients with clinically and pathologically confirmed PD and from those without PD (control samples) were obtained from the Research Resource Network through the National Center of Neurology and Psychiatry, Musashi Hospital (Japan). The present study was approved by the Institutional Review Board (IRB) of the Ajou University Medical Center (AJOUIRB-KSP-2020-587) for the use of postmortem human brain tissues. Information regarding the donors is provided in Supplementary Table S1.

### Immunohistochemistry

Paraffin-embedded 6-μm-thick brain tissue sections were deparaffinised thrice in xylene for 5 min. The samples were rehydrated using a graded ethanol series (100%, 95%, and 70%) for 3 min, followed by washing in distilled water for 5 min. Endogenous peroxidase activity was quenched by incubating with Peroxidase-Blocking Solution (Agilent Dako, Glostrup, Denmark) for 10 min. After washing twice with PBS, the sections were incubated for 16 h at 4°C with primary antibodies against synphilin-1 (NBP2-55955; Novus Biologicals, Littleton, CO, U.S.A.), tyrosine hydroxylase (TH) (sc-14007; Santa Cruz Biotechnology), and p53 (sc-126; Santa Cruz Biotechnology) diluted 1:100 in antibody diluent (Agilent Dako). Then, the sections were washed thrice with wash buffer and incubated with the HRP-labelled polymer (Agilent Dako) for 30 min at 25°C. After additional washes with the wash buffer, the immunoreactive signals were visualised by treating with the chromogen 3,3′-diaminobenzidine tetrahydrochloride for 3 min, followed by rinsing with distilled water for 3 min. The sections were counterstained with haematoxylin for 3 min, rinsed with distilled water, dehydrated through a graded ethanol series, cleared in xylene, and mounted using Tissue-Tek SGC400 (Sakura Finetek Japan, Tokyo, Japan). Images were acquired using a slide scanner (EasyScan Pro 6; Motic) and analysed using Motic DSAssistant software.

### Cell viability assay

Cell viability was assessed using the Cell Proliferation Kit I [MTT (3-[4,5-dimethylthiazol-2-yl]-2,5-diphenyltetrazolium bromide)] (Roche, Basel, Switzerland), according to the manufacturer’s instructions. Cells were seeded onto 96-well plates and incubated at 37°C. MTT assays were performed based on the experimental design, and MTT labelling reagent was added to each well, followed by incubation for 2 h at 37°C. Then, the solubilisation solution was added and incubated for 16 h at 37°C. Absorbance was measured at 570 nm (measurement wavelength) and 690 nm (reference wavelength) using a microplate spectrophotometer (Epoch 2; BioTek, Winooski, VT, U.S.A.).

### Statistical analysis

Statistical analyses were performed using IBM SPSS Statistics 20 (IBM Corporation, Armonk, NY, U.S.A.). Differences between two independent groups were analysed using an independent-samples *t*-test after confirming homogeneity of variances with Levene’s test. Statistical significance was defined as *P* < 0.05. The sample sizes (*n*) for each analysis are included in the corresponding figure legends, and the *P*-values are reported in the figures.

## Results

### Integrated transcriptomic and proteomic analyses show morphological alterations and anoikis resistance in synphilin-1-overexpressing cells

**Figure 1:**
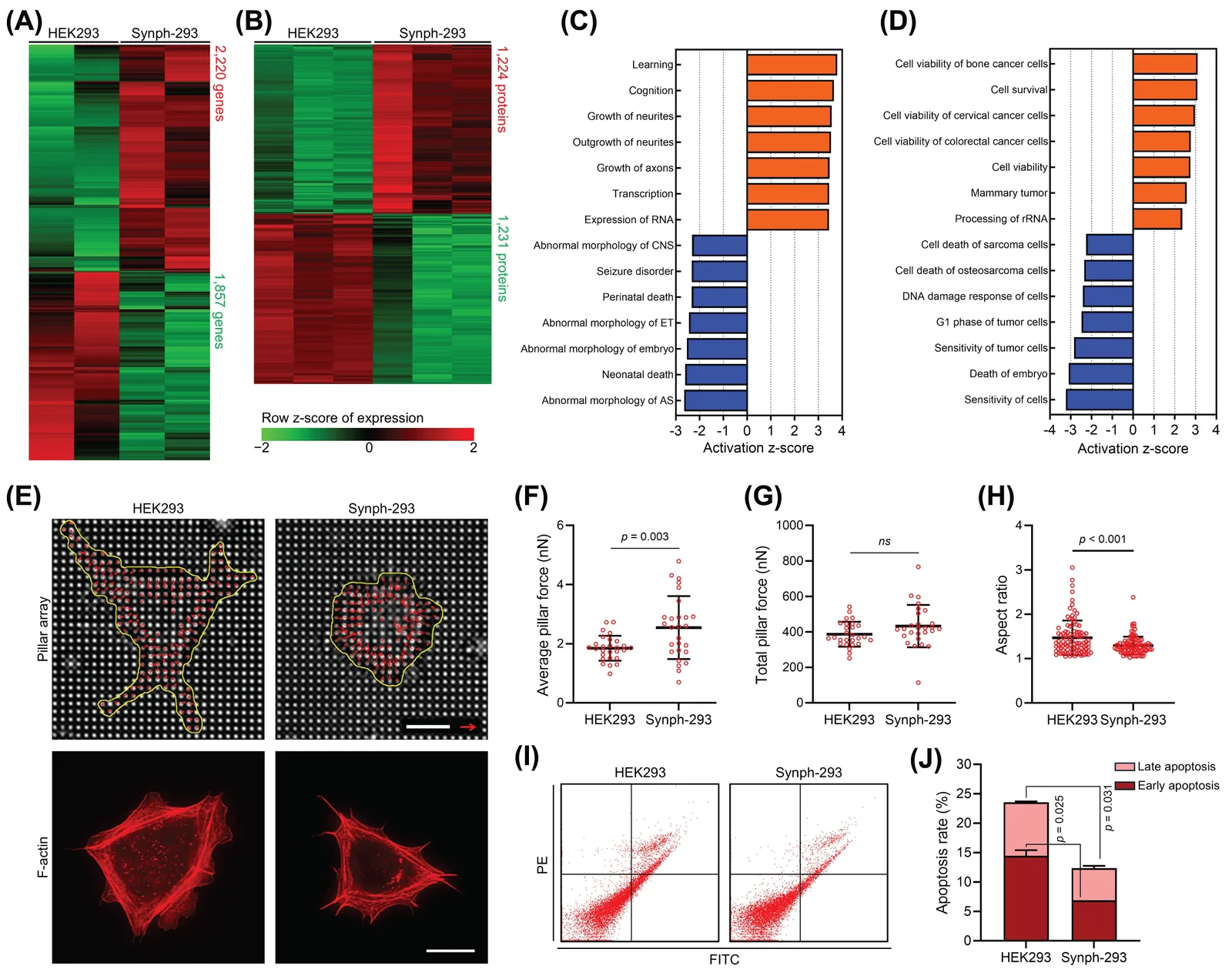
Transcriptomic and proteomic analyses reveal altered cellular morphology and anoikis resistance in synphilin-1-overexpressing cells (**A**) Heatmap of differentially expressed gene profiles in HEK293 and synphilin-1-overexpressing HEK293 (Synph-293) cells. (**B**) Heatmap of differentially expressed protein profiles in HEK293 and Synph-293 cells. (**C**) Transcriptomic analysis of the top seven activated or inhibited diseases and biological functions predicted using IPA. CNS: central nervous system; ET: embryonic tissue; AS: axial skeleton. (**D**) Proteomic analysis of the top seven activated or inhibited diseases and biological functions. (**E**) Pillar deflection maps and F-actin staining images of HEK293 and Synph-293 cells after 1 h of incubation on pillar arrays and glass substrates, respectively (scale bar = 10 μm, scale arrow = 10 nN for pillar array; scale bar = 20 μm for F-actin staining). (**F**) Average traction force and (**G**) total traction force of pillars beneath HEK293 and Synph-293 cells (*n* = 27). (**H**) Cell aspect ratio of cells (*n* = 100). Graphs represent the mean ± standard deviation (SD). (**I**) Flow cytometric analysis and (**J**) quantification of apoptosis rates of HEK293 and Synph-293 cells cultured on low-binding substrates (*n* = 2). Graphs represent the mean + SD. Statistical significance was determined using an independent sample *t*-test and Levene’s test.

We investigated the effect of synphilin-1 overexpression by performing integrated transcriptomic and proteomic analyses using HEK293 and Synph-293 cells. Synphilin-1 expression in Synph-293 cells was confirmed at both the transcriptomic and proteomic levels (Supplementary Figure S1A,B). Transcriptomic and proteomic profiling showed extensive changes in gene and protein expression of Synph-293 cells compared with those in HEK293 cells (Figure 1A,B). Cellular functional analysis of the differentially expressed genes using the ingenuity pathway analysis (IPA) bioinformatics tool predicted the activation of cell morphology-associated functions such as neurite and axon growth; in contrast, functions associated with abnormal morphologies were inhibited (Figure 1C). IPA analysis of the proteomic changes showed enhanced cell viability-associated functions and suppressed cell death-related functions (Figure 1D). These results suggest that synphilin-1 overexpression promotes cell survival and morphological changes in HEK293 cells.

Next, we examined the biophysical and viability properties of Synph-293 cells. As early cell adhesion and signalling induce survival pathways that inhibit cell death [27,28], we analysed the attachment to the pillar arrays and coverslips during the first hour. Cellular force measurement using an elastomeric pillar array showed that Synph-293 cells induced a significant central deflection of the underlying pillars. Similarly, the Synph-293 cells exhibited a contracted morphology on the coverslip (Figure 1E). Quantification of the pillar forces showed that the average force of the Synph-293 cells on each pillar was significantly higher than that of the HEK293 cells (Figure 1F), although the total force applied to all pillars beneath each cell did not change significantly (Figure 1G). The aspect ratio of Synph-293 cells on the coverslip was close to 1 compared with that observed for the HEK293 cells (Figure 1H), which implies that Synph-293 cells exhibited a contracted morphology. At a later time point, the Synph-293 cells exhibited a contracted stellate morphology, whereas the HEK293 cells exhibited a more spread-out shape (Supplementary Figure S1C). Upon synphilin-1 knockdown, Synph-293 cells regained a rounded morphology similar to that of the HEK293 cells (Supplementary Figure S1D,E). Based on the proteomic prediction of enhanced cell viability and *in vitro* results for Synph-293 cell phenotypes, we hypothesised that Synph-293 cells may be resistant to anoikis. Indeed, compared with HEK293 cells, the Synph-293 cells exhibited a significant decrease in apoptosis when cultured on low-binding plates and analysed by flow cytometry (Figure 1I,J). These findings demonstrate that synphilin-1 overexpression induces a more contractile cell morphology and suppresses anoikis under reduced adhesion conditions.

**Figure 2:**
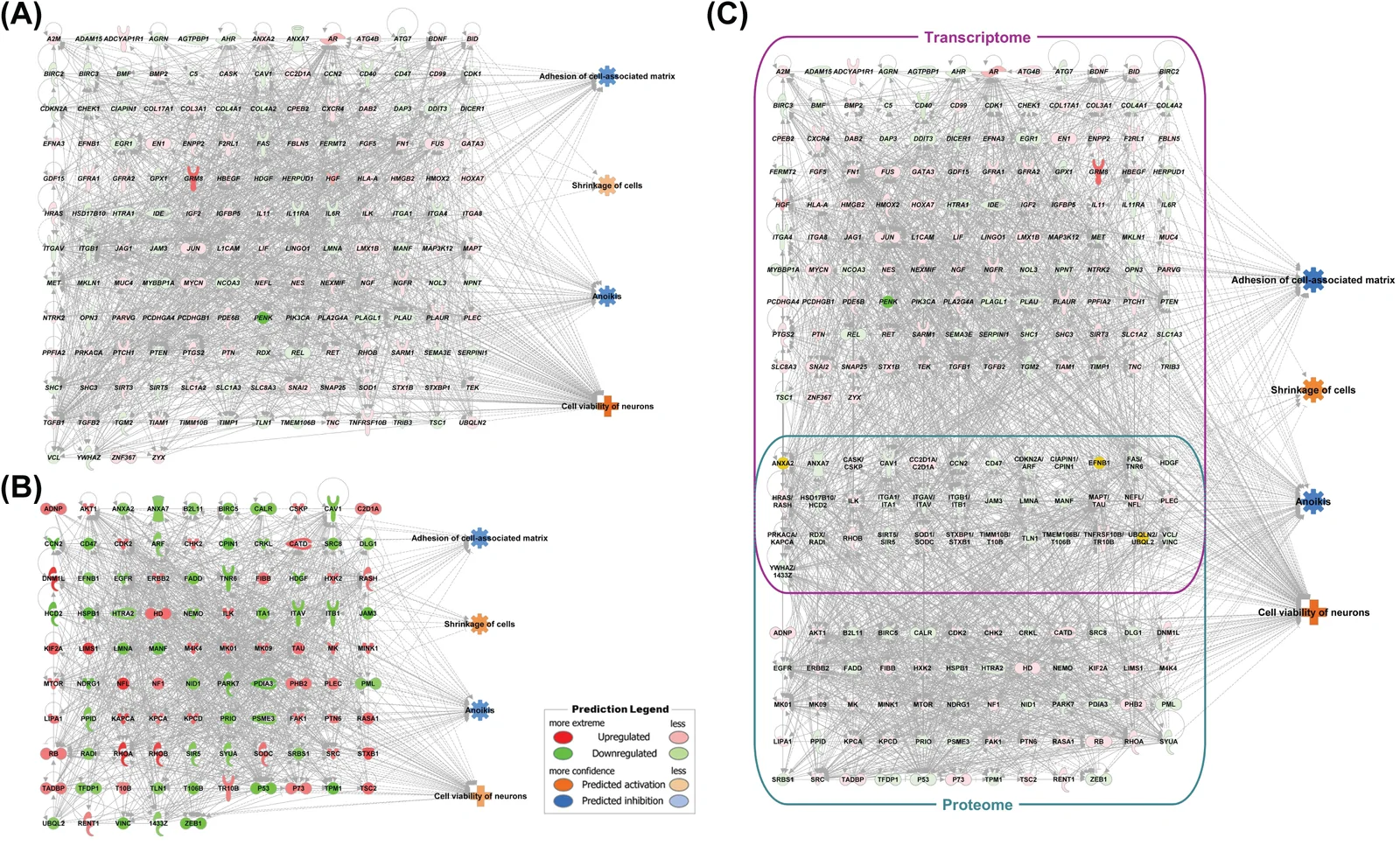
Transcriptomic, proteomic, and integrated omics network analyses predict functional alterations in synphilin-1-overexpressing cells (**A**) Transcriptomic, (**B**) proteomic, and (**C**) integrated omics networks with *in silico* prediction of Synph-293 cell functions using IPA. Molecules with opposite expressions are indicated in yellow.

To validate the phenotypic observations, functional networks were constructed using transcriptomic, proteomic, and integrated datasets. IPA-based networks focusing on the adhesion of cells to the associated matrix, shrinkage of cells, anoikis, and neuron viability showed coherent prediction trends across all three datasets (Figure 2). The transcriptome-derived network predicted the inhibition of cell-associated matrix adhesion and anoikis and the activation of cell shrinkage and neuron viability (Figure 2A and Supplementary Figure S2A). The proteome-based network provided similar predictions (Figure 2B and Supplementary Figure S2B). The expression and molecular details of the IPA network-associated genes and proteins are summarised in Supplementary Tables S2 and S3, respectively. Integration of the transcriptomic and proteomic datasets strengthened the overall interpretation by linking gene- and protein-level alterations underlying the synphilin-1 overexpression-mediated phenotypes (Figure 2C and Supplementary Figure S2C). In particular, seven molecules linked to the inhibition of anoikis (*CDKN2A, FAS, HRAS, ILK, ITGAV, PLEC*, and *RDX*) showed consistent alterations in both gene and protein expression levels (Supplementary Figure S2D,E). In summary, multi-omics and experimental validation collectively suggest that synphilin-1 overexpression promotes contractile morphology, protects against anoikis, and establishes a cell-survival-promoting molecular program.

### Synphilin-1 down-regulates p53 and suppresses anoikis in neuronal cells, and p53 nuclear localisation is observed in tissues from patients with Parkinson’s disease

**Figure 3:**
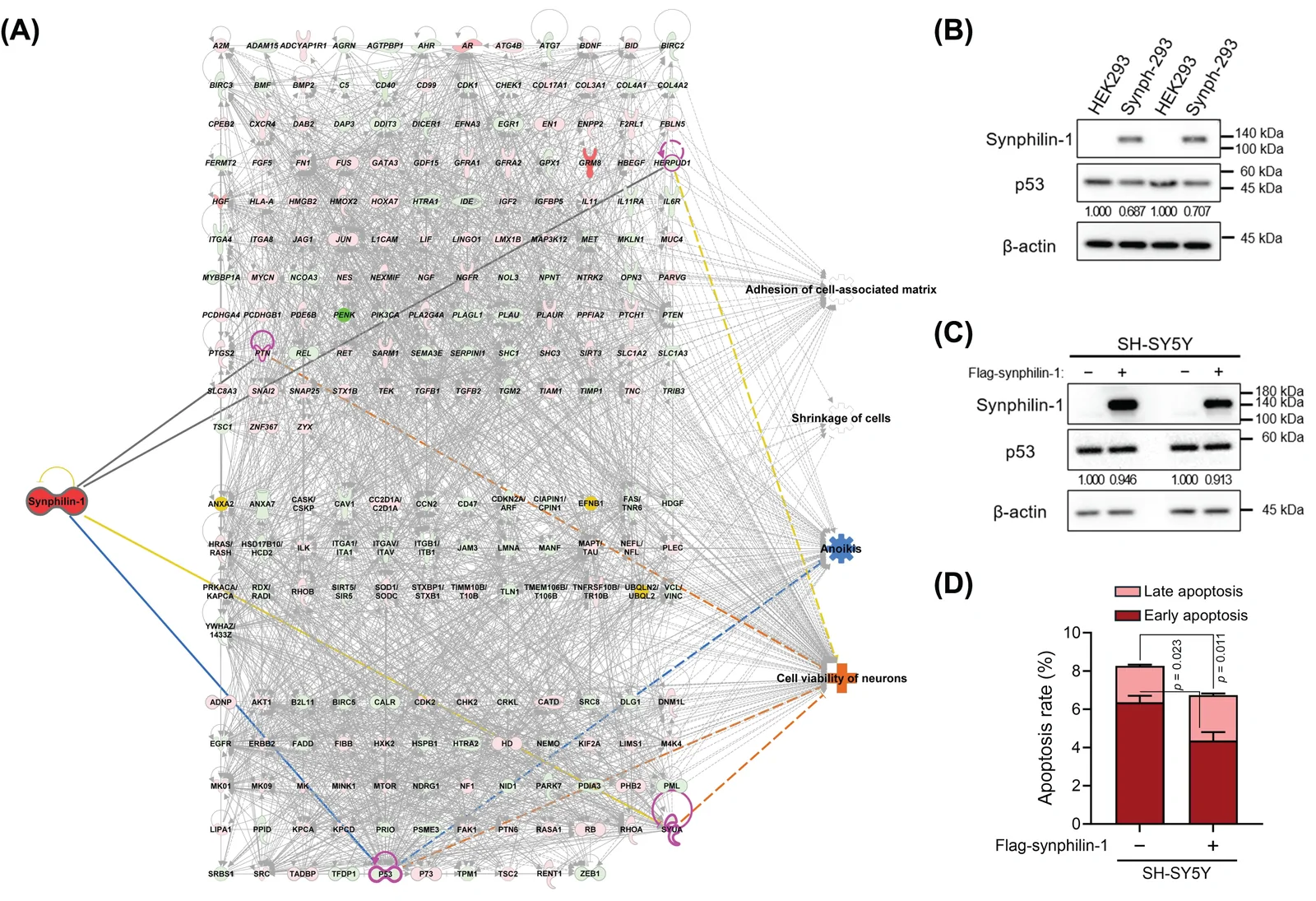
Synphilin-1 down-regulates p53 and suppresses anoikis in neuronal cells (**A**) IPA network of association between synphilin-1 and related molecules identified from the integrated omics network. Blue and orange, respectively, represent the inhibition and activation of molecules and cellular functions. Molecules and lines with opposite expressions or predictions are indicated in yellow. (**B**) Western blotting analysis of p53 expression in HEK293 and Synph-293 cells (*n* = 2). (**C**) Western blotting analysis of p53 expression in SH-SY5Y cells transiently overexpressing synphilin-1 (*n* = 2). (**D**) Flow cytometric analysis of apoptosis rates in SH-SY5Y cells transiently overexpressing synphilin-1 (*n* = 3). Graphs represent the mean + SD. Statistical significance was determined using an independent sample *t*-test and Levene’s test.

To further identify the molecular mediators of synphilin-1-dependent anoikis suppression, we examined the integrated omics network for direct connections with synphilin-1. We found molecular connections between synphilin-1 and *HERPUD1, PTN*, SYUA, and p53 (Figure 3A and Supplementary Figure S3A). Among these, p53 was identified as the key node that was negatively regulated by synphilin-1. Consistently, correlation analyses revealed a significant negative correlation between synphilin-1 and p53 expression in both the Synph-293 proteomic dataset and a dopaminergic neuron transcriptomic GEO dataset (GSE169755) [29] (Supplementary Figure S3B,C). This regulation was predicted to inhibit anoikis and activate neuronal viability. Based on this network prediction, we selected p53 as the candidate effector that mediates the protective function of synphilin-1. Immunoblotting analysis distinctly indicated a decrease in p53 levels in Synph-293 cells compared with those in HEK293 cells (Figure 3B). To confirm this effect in neuronal cells, SH-SY5Y cells were transiently transfected with Flag-synphilin-1. Consistently, synphilin-1 overexpression decreased p53 protein levels in SH-SY5Y cells (Figure 3C). Functionally, transient synphilin-1 overexpression in SH-SY5Y cells suppressed anoikis in the low-binding plates (Figure 3D). This finding indicates that synphilin-1 down-regulates p53 expression, thereby protecting against anoikis, supporting its protective effect in neuronal cells.

**Figure 4:**
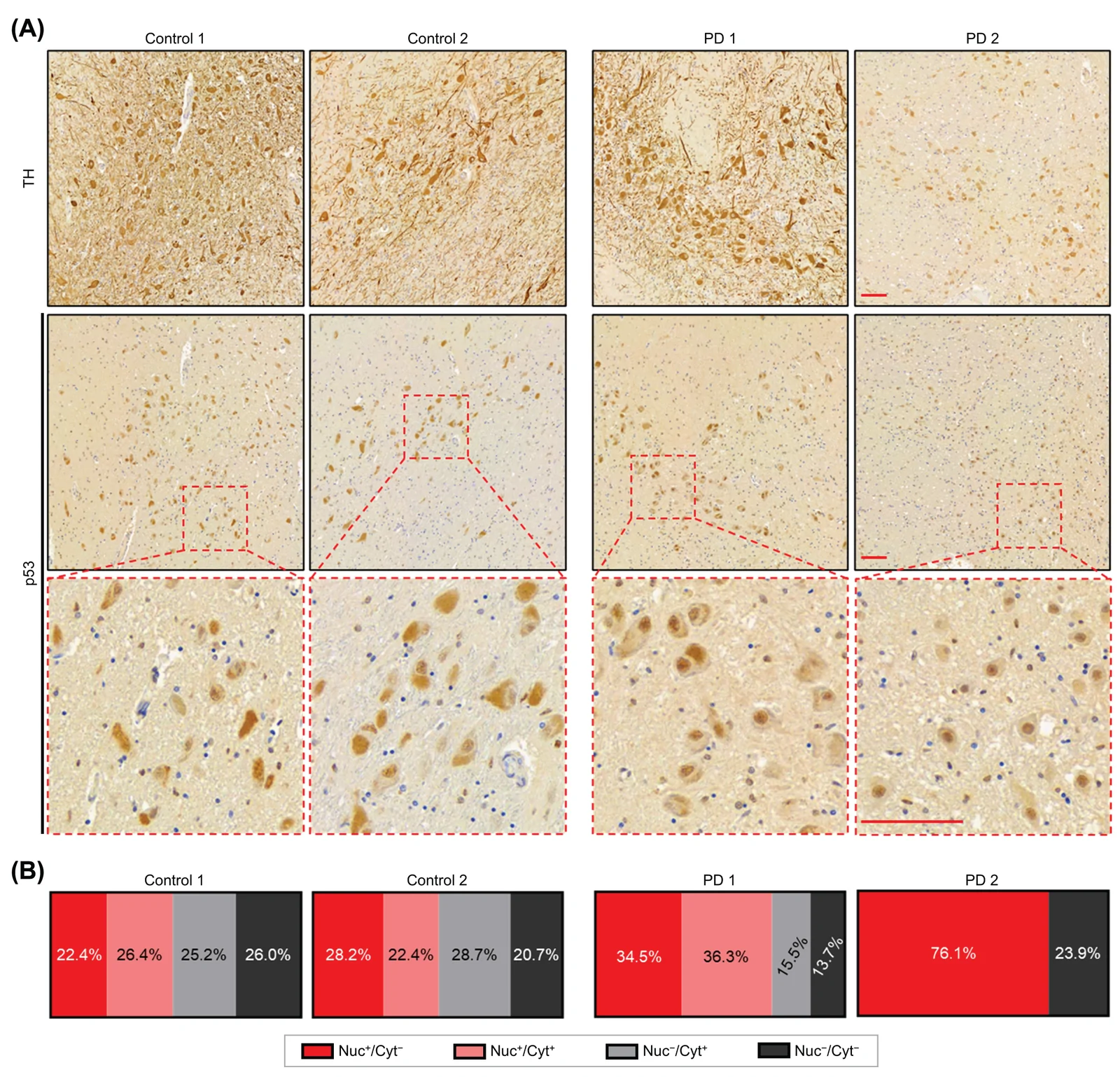
Altered p53 subcellular localisation in dopaminergic neurons of patients with PD (**A**) Immunohistochemical analysis of TH and p53 expression in postmortem midbrain samples from patients with and without (control) PD (scale bars = 100 μm). (**B**) Quantification of p53 expression patterns categorised based on nuclear (Nuc) and cytoplasmic (Cyt) localisation in postmortem midbrain samples from patients with and without (control) PD.

Subsequently, we identified the clinical relevance of p53 expression by examining the p53 expression pattern in postmortem human midbrain tissues obtained from two patients with PD and two control individuals (Supplementary Figure S4). Immunohistochemistry with TH and p53 showed prominent nuclear accumulation of p53 in the dopaminergic neurons of the PD samples in contrast to its cytoplasmic distribution in the control samples (Figure 4A). The quantitative classification of the p53 staining patterns into four categories showed a pronounced increase in nuclear-positive populations in the PD tissues (Figure 4B). In the TH-positive dopaminergic neuron regions, synphilin-1 and TH expression did not directly indicate differences between PD and control samples (Supplementary Figure S5); however, p53 exhibited distinct subcellular localisation with pronounced nuclear localisation in PD tissues. Specifically, in control tissues, the proportions of nuclear-positive cells were 48.8% and 50.6%, respectively. In PD tissues, the corresponding proportions were 70.8% and 76.1%, respectively.

Additionally, cell viability after dose-dependent 1-methyl-4-phenylpyridinium (MPP^+^) exposure, which is widely used as a cellular model of PD, showed that synphilin-1 overexpression enhanced cell viability, whereas synphilin-1 knockdown reduced viability (Supplementary Figure S6A–D). Furthermore, p53 knockdown alleviated MPP^+^-induced toxicity, mitigating the reduction in cell viability (Supplementary Figure S6E,F). This result supports the protective role of synphilin-1 against PD-related cellular stress. Taken together, these findings show that synphilin-1 overexpression suppresses p53 expression and anoikis. The nuclear localisation of p53 in the PD midbrain samples suggests that synphilin-1 overexpression may exert a protective effect through p53 regulation.

## Discussion

Previous studies have demonstrated that synphilin-1 exerts its neuroprotective effects through multiple cellular mechanisms. Synphilin-1 interacts with and activates AMP-activated protein kinase, enhancing ATP production and maintaining energy homeostasis [30]. In neuronal cells, synphilin-1 overexpression mitigated MPP^+^- and rotenone-induced toxicity by reducing the production of reactive oxygen species, activated extracellular signal-regulated kinases, and inhibited caspase-3 and poly ADP-ribose polymerase cleavage [3,4]. Furthermore, synphilin-1 attenuated toxicity in PD models expressing mutant α-synuclein and leucine-rich repeat kinase 2 [5–7]. Notably, synphilin-1 suppressed the transcriptional activity and expression of p53, thereby exerting anti-apoptotic effects [31]. Collectively, these findings suggest that synphilin-1 functions as a multifaceted neuroprotective regulator by modulating various cellular pathways.

Recently, ECM remodelling and adhesion-dependent signalling have been suggested to contribute to PD pathology. Huang et al*.* demonstrated that 1-methyl-4-phenyl-1,2,3,6-tetrahydropyridine treatment-induced fibronectin accumulation exacerbated α-synuclein aggregation through integrin α4β1-mediated signalling [32]. This indicates a role for aberrant cell–ECM interaction in neuronal stress. Additionally, ECM remodelling alters its composition and tissue stiffness, which dysregulates integrin-mediated adhesion and focal adhesion signalling that are essential for neuronal survival in brain diseases [33]. Consistent with the identification of anoikis-related gene signatures in PD [15], these findings suggest that disrupted adhesion signalling may influence neuronal vulnerability and survival.

We have previously shown that synphilin-1 overexpression impairs cellular rigidity sensing on substrates [22]. Mechanical insensitivity caused by synphilin-1 overexpression resembles the phenotype observed in transformed cancer cells that have lost their rigidity-dependent growth and acquired resistance to anoikis [9,34]. This suggests that synphilin-1 possibly affects the mechanotransduction pathways that connect ECM stiffness and anoikis.

In addition, we previously performed traction force analyses using a dense pillar array (500 nm diameter, 1 μm spacing) and showed that Synph-293 cells exhibited higher average but lower total traction forces than those of HEK293 cells [22]. Although a platform with a larger pillar diameter and wider spacing (900 nm diameter, 1.8 μm spacing) was used in the present study, the average traction force was found to similarly increase, whereas the total force exhibited no significance. These results indicate that synphilin-1 overexpression enhanced cell contractility regardless of the pillar geometry, which suggests that its effect reflects an intrinsic mechanical phenotype rather than a pillar array design.

The present study has some limitations that need to be acknowledged. First, the immunohistochemical analysis was performed on a limited number of human midbrain samples, which restricts the reliability of the observed p53 localisation patterns. Second, no significant difference in synphilin-1 expression was observed between the PD and control tissue samples, suggesting that our observations primarily highlight the potential neuroprotective effects of synphilin-1 overexpression rather than its endogenous dysregulation in PD. Third, most of our *in vitro* experiments relied on the HEK293 and Synph-293 cells, which do not fully reflect the physiological properties of dopaminergic neurons, although key findings were validated in SH-SY5Y cells. Finally, while our integrated omics and IPA approach provided a comprehensive overview, it remains a predictive analysis that simplifies a vast scale of molecular alterations. Consequently, this single synphilin-1–p53 axis may not fully capture the complex regulatory mechanisms operating within the massive multi-omics datasets.

## Future directions

Future studies should validate the pathological relevance of p53 dysregulation in larger cohorts of patients with PD. To establish a causal and bidirectional relationship within the synphilin-1–p53 axis, future investigations should explore the effects of p53 knockdown or overexpression on synphilin-1 expression, alongside p53 rescue experiments to confirm the underlying protective mechanism. Furthermore, future *in vitro* studies should expand beyond HEK293 cells to incorporate other human neuronal cell lines. Ultimately, the use of induced pluripotent stem cell-derived dopaminergic neurons and other robust human neuronal models will be essential to clarify the mechanisms through which the synphilin-1–p53 axis contributes to neuronal resilience in PD. Expanding the multi-omics approach to include additional layers, such as phosphoproteomics and metabolomics, may provide deeper mechanistic insight.

## Supplementary Material

Supplementary Figures S1-S6 and Tables S1-S3

## Data Availability

The data supporting the results of the present study are available from the corresponding author upon reasonable request. Transcriptome sequencing and quantification data have been deposited in the GEO database (accession number: GSE255201) [35], and the proteome data are available from the MassIVE database (accession number: MSV000089926) [36].

## Abbreviations

DMEM: Dulbecco’s Modified Eagle Medium
ECM: extracellular matrix
FBS: foetal bovine serum
FDR: false discovery rate
FITC: fluorescein isothiocyanate
GEO: Gene Expression Omnibus
HEK293: human embryonic kidney 293
*HERPUD1*: homocysteine inducible ER protein with ubiquitin like domain 1
HRP: horseradish peroxidase
IPA: Ingenuity Pathway Analysis
IRB: Institutional Review Board
MPP^+^: 1-methyl-4-phenylpyridinium
MTT: 3-(4,5-dimethylthiazol-2-yl)-2,5-diphenyltetrazolium bromide
NF: neurofilament
PBS: phosphate-buffered saline
PD: Parkinson’s disease
PDMS: polydimethylsiloxane
*PTN*: pleiotrophin
PVDF: polyvinylidene difluoride
SD: standard deviation
Synph-293: HEK293 cell line that stably expresses synphilin-1
SYUA: α-synuclein
TH: tyrosine hydroxylase
TMT: tandem mass tag
