# Supplementary material for: Integrated omics-driven discovery reveals synphilin-1-mediated anoikis resistance through p53 down-regulation
Source: Biosci Rep. 2026 Sep 18;46(10):BSR20260173. doi: 10.1042/BSR20260173 (PMC13591246; doi:10.1042/BSR20260173)
Supplement: Supplementary file 1 [file BSR-2026-0173_supp.pdf]

Supplementary data

Integrated Omics-Driven Discovery Reveals Synphilin-1-mediated Anoikis Resistance through p53 Downregulation

Seok Gi Kim[1†], Ye Eun Sim[2†], Abdurazak Aman Ketebo[3†], Ji Su Hwang[1,4], Nimisha Pradeep George[4], Yong Eun Jang[1,4], Minjun Kwon[1], Sungsu Park[3,5,6], Jung-Soon Mo[2,7*], Gwang Lee[1,4*]*

[1] Department of Physiology, Ajou University School of Medicine, 164 World Cup-ro, Suwon 16499, Republic of Korea.

[2] Department of Biomedical Sciences, BK21 R&E Initiative for Advanced Precision Medicine, Graduate School of Ajou University, 164 World cup-ro, Suwon 16499, Republic of Korea.

[3] Department of Mechanical Engineering, Sungkyunkwan University, 2066 Seobu-ro, Suwon 16419, Republic of Korea.

[4] Department of Molecular Science and Technology, Ajou University, 206 World cup-ro, Suwon 16499, Republic of Korea.

[5] Institute of Quantum Biophysics (IQB), Sungkyunkwan University, 2066 Seobu-ro, Suwon 16419, Republic of Korea.

[6] Department of MetaBioHealth, Sungkyunkwan University, 2066 Seobu-ro, Suwon 16419, Republic of Korea.

[7] Institute of Medical Science, Ajou University School of Medicine, 164 World cup-ro, Suwon 16499, Republic of Korea.

[†]These authors contributed equally to this work.

[*]Corresponding authors.

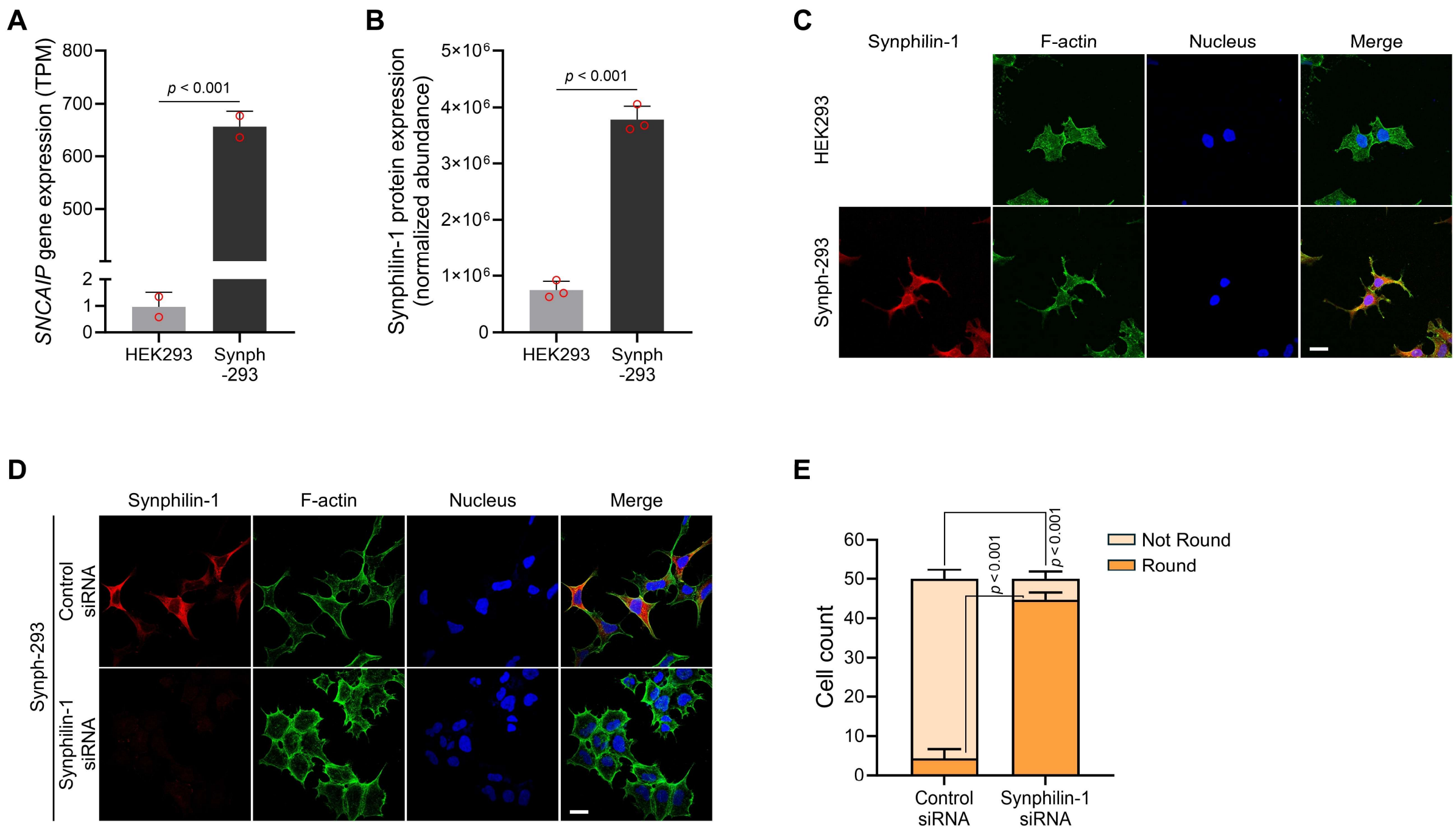

Supplementary Figure 1: **Synphilin-1 expression and cell morphological changes in synphilin-1-overexpressing cells.**

(A) Gene expression levels of synphilin-1 (*SNCAIP*) in HEK293 and synphilin-1-overexpressing HEK293 (Synph-293) cells based on transcriptomic data. (B) Protein expression levels of synphilin-1 in HEK293 and Synph-293 cells based on the proteomic data. (C) Fluorescence staining of synphilin-1 and F-actin in HEK293 and Synph-293 cells (scale bar = 20 μm). (D) Fluorescence images of Synph-293 cells with synphilin-1 knockdown (scale bar = 20 μm). (E) Quantification of cell morphology in Synph-293 cells with synphilin-1 knockdown (n = 50). Graphs represent the mean + standard deviation (SD). Statistical significance was determined using an independent sample t-test with Levene's test.

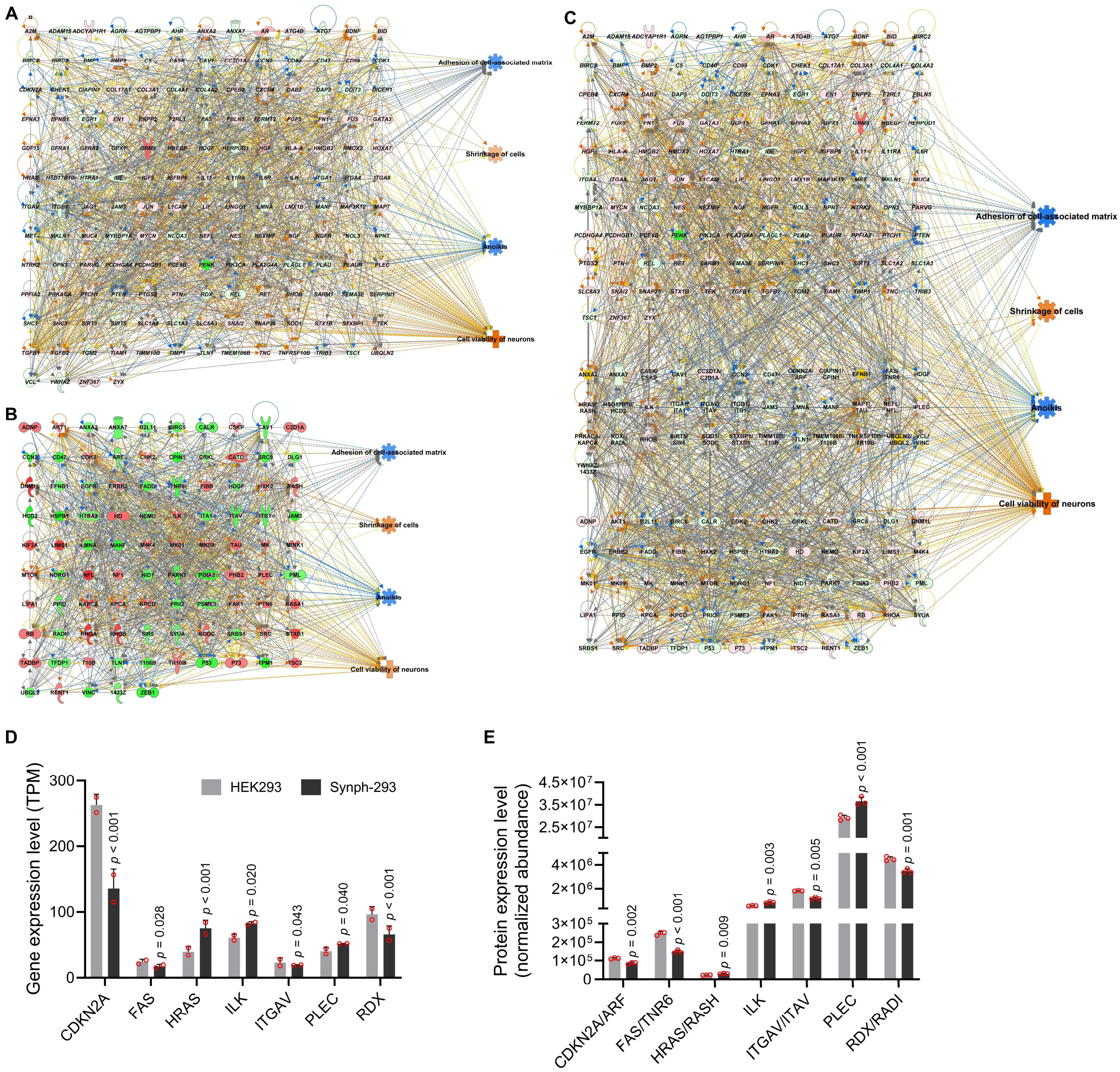

Supplementary Figure 2: **Transcriptomic, proteomic, and integrated omics networks predicting the Synph-293 cell phenotype.**

(A) Transcriptomic, (B) proteomic, and (C) integrated omics networks with an *in silico* prediction of Synph-293 cell functions using Ingenuity Pathway Analysis (IPA). Molecules with opposite expressions are indicated in yellow. (D) Transcriptomic expression profiles and (E) proteomic expression profiles obtained from MS/MS analysis of seven selected molecules predicted to inhibit anoikis, showing consistent expression changes.

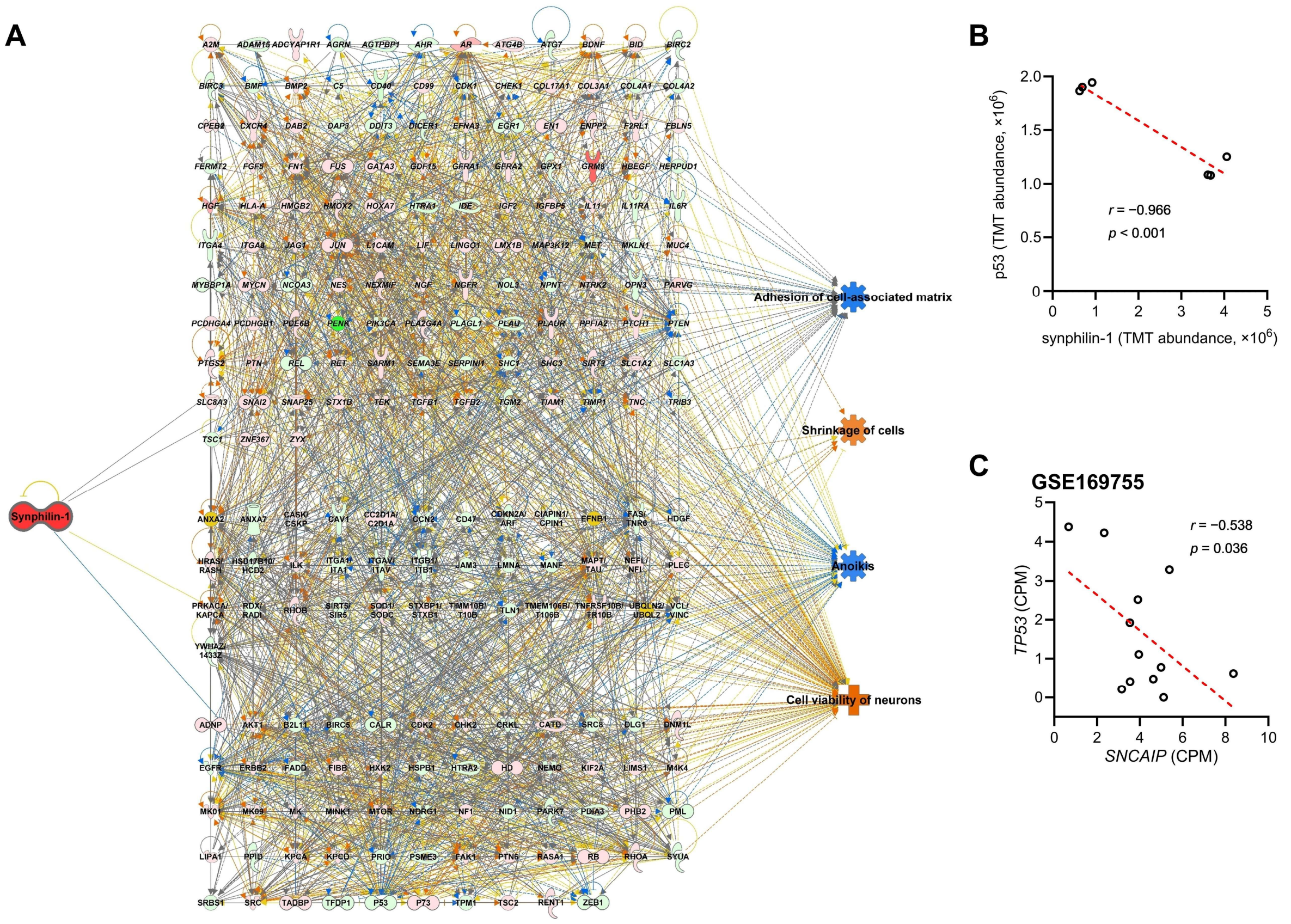

Supplementary Figure 3: **Integrated omics network and correlation analyses of synphilin-1 with related molecules.**

(A) IPA network of the association between synphilin-1 and related molecules identified from the integrated omics network. (B) Correlation between synphilin-1 and p53 expression from proteomic dataset. (C) Correlation between SNCAIP (synphilin-1 gene) and TP53 (p53 gene) expression in dopaminergic neurons from the GSE169755 dataset. The linear regression line is indicated as a red dashed line. Pearson's correlation coefficient (*r*) was calculated using a one-tailed significance test.

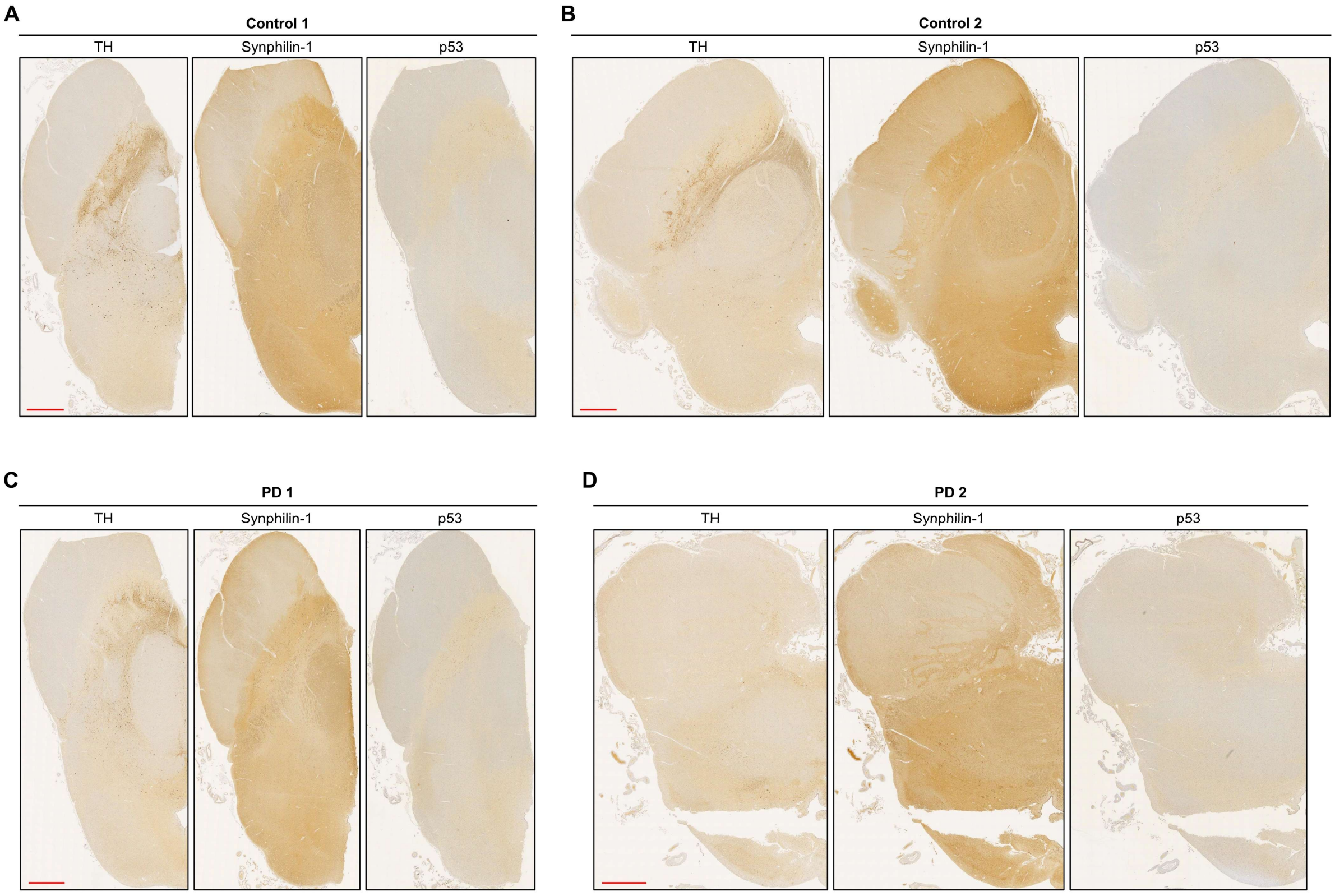

Supplementary Figure 4: Immunohistochemical staining of midbrain tissues from control and patients with Parkinson's disease.

(A–D) Immunohistochemical images of tyrosine hydroxylase (TH), synphilin-1, and p53 in entire midbrain hemispheres from Control 1 (A), Control 2 (B), PD 1 (C), and PD 2 (D) (scale bars = 2 mm).

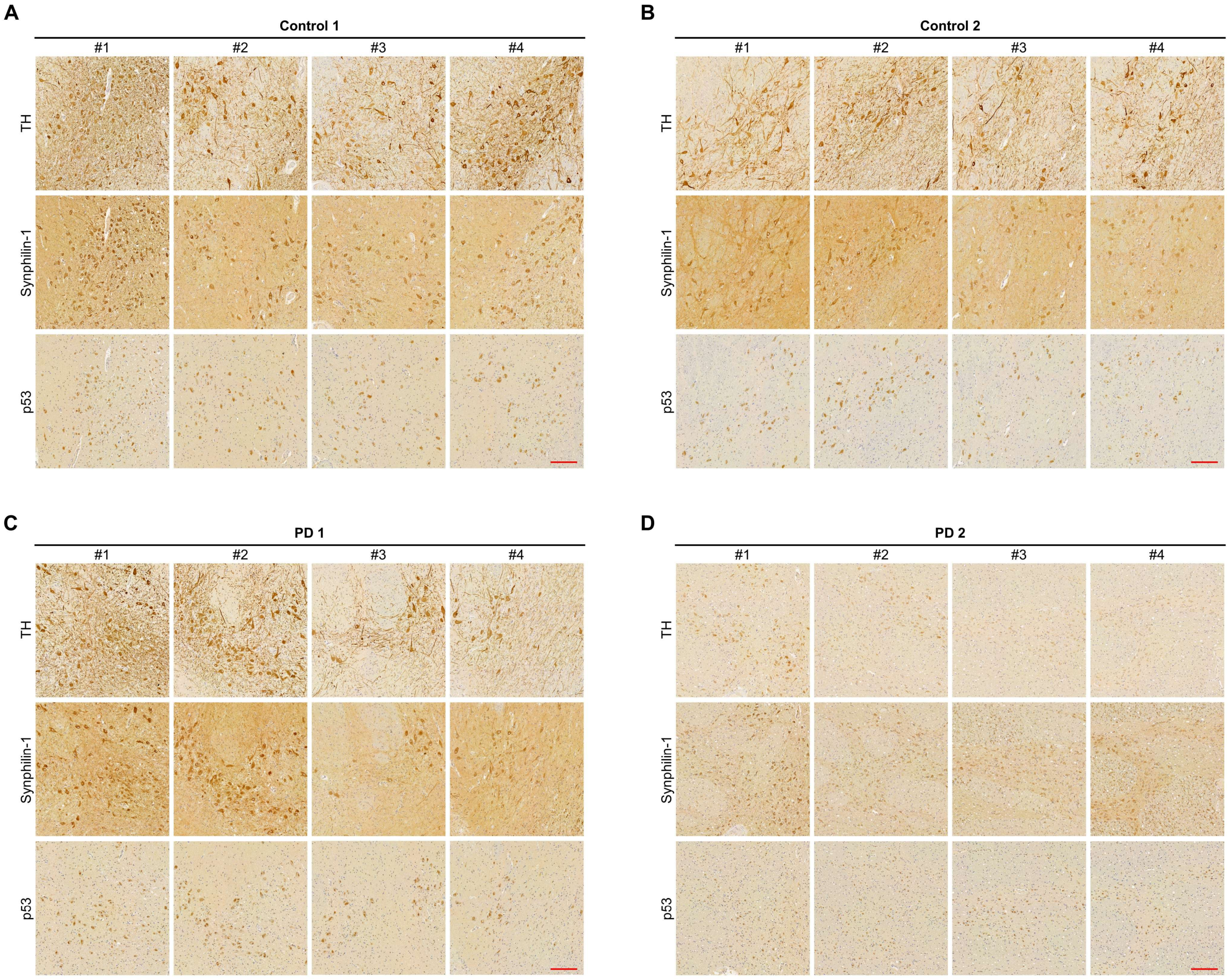

Supplementary Figure 5: High magnification immunohistochemical images of midbrain tissues from control and patients with Parkinson’s disease.

(A–D) High magnification immunohistochemical images of TH, synphilin-1, and p53 in midbrain tissues from Control 1 (A), Control 2 (B), PD 1 (C), and PD 2 (D) (scale bars = 200 μm). The images correspond to the four regions used for p53 analysis in Figure 4B.

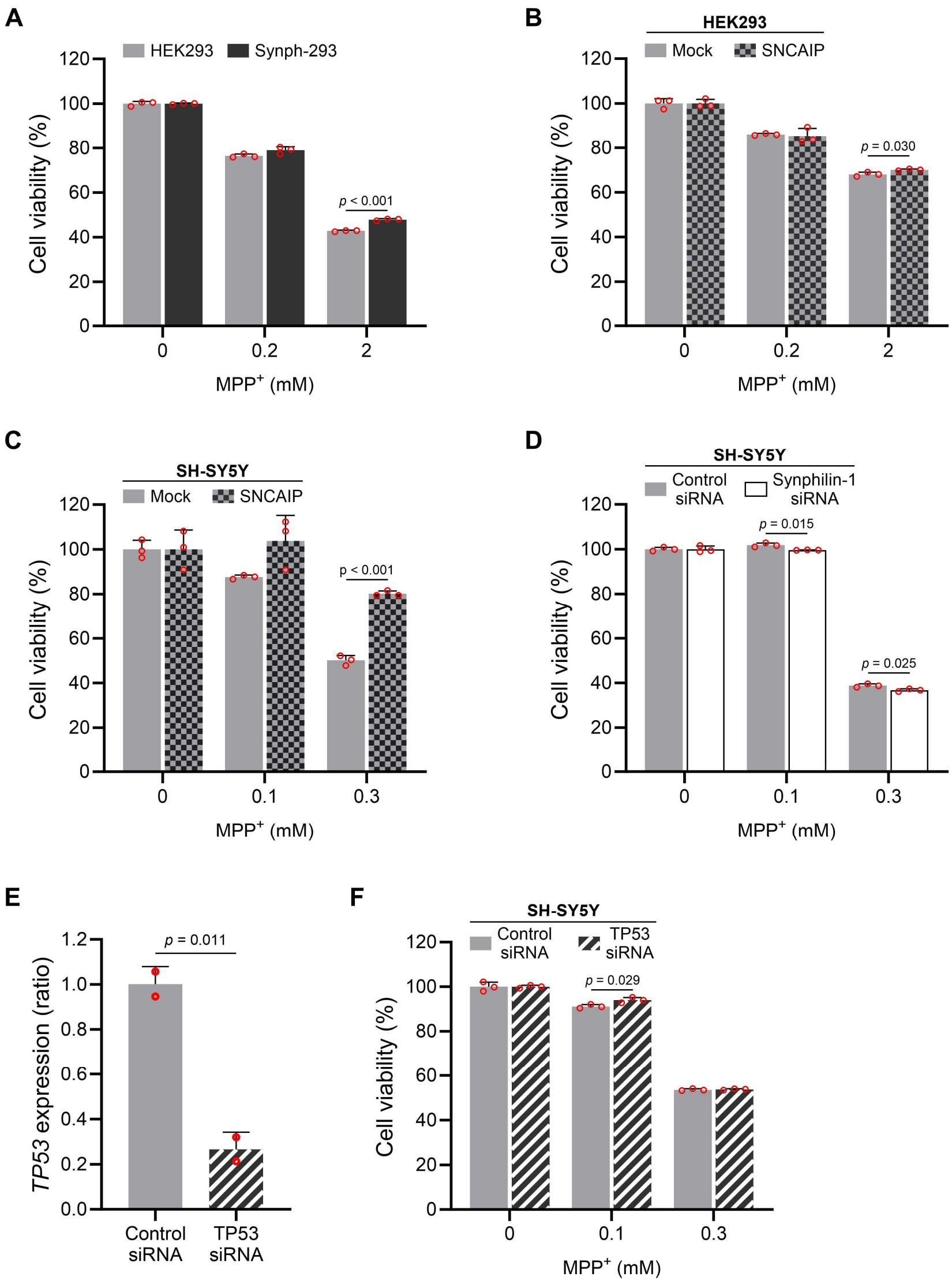

Supplementary Figure 6: Effects of synphilin-1 and p53 expression on 1-methyl-4-phenylpyridinium (MPP$^+$)-induced cytotoxicity in cells.

(A) Cell viability of HEK293 and Synph-293 cells following MPP$^+$ treatment for 24 h. (B) Cell viability of HEK293 cells transfected with synphilin-1 following MPP$^+$ treatment for 24 h. (C) Cell viability of SH-SY5Y cells transfected with synphilin-1 following MPP$^+$ treatment for 24 h. (D) Cell viability of SH-SY5Y cells with synphilin-1 knockdown following MPP$^+$ treatment for 24 h. (E) *TP53* (p53) gene expression in SH-SY5Y cells transfected with *TP53* siRNA. (F) Cell viability of SH-SY5Y cells with p53 knockdown following MPP$^+$ treatment for 24 h. Graphs represent the mean + SD. Statistical significance was determined using an independent sample *t*-test with Levene's test.

Supplementary Table 1: Information on postmortem midbrain samples

Sample name	Diagnosis	Sex	Age (years)
Control 1	Schizophrenia (non-Parkinson control)	Male	73
Control 2	Schizophrenia (non-Parkinson control)	Male	73
PD 1	Parkinson’s disease	Female	83
PD 2	Parkinson’s disease	Female	76

Supplementary Table 2: Profiles of the genes in the Ingenuity Pathway Analysis-based transcriptomic network

Gene name	Symbol	Entrez Gene ID	Fold change
alpha-2-macroglobulin	*A2M*	2	9.71
ADAM metallopeptidase domain 15	*ADAM15*	8751	-1.64
ADCYAP receptor type I	*ADCYAP1R1*	117	9.10
agrin	*AGRN*	375790	-1.47
ATP/GTP binding carboxypeptidase 1	*AGTPBP1*	23287	-1.45
aryl hydrocarbon receptor	*AHR*	196	-1.43
annexin A2	*ANXA2*	302	1.31
annexin A7	*ANXA7*	310	-1.43
androgen receptor	*AR*	367	34.31
autophagy related 4B cysteine peptidase	*ATG4B*	23192	1.45
autophagy related 7	*ATG7*	10533	-1.57
brain derived neurotrophic factor	*BDNF*	627	1.61
BH3 interacting domain death agonist	*BID*	637	1.46
baculoviral IAP repeat containing 2	*BIRC2*	329	-1.45
baculoviral IAP repeat containing 3	*BIRC3*	330	-2.85
Bcl2 modifying factor	*BMF*	90427	-1.87
bone morphogenetic protein 2	*BMP2*	650	11.42
complement C5	*C5*	727	-2.60
calcium/calmodulin dependent serine protein kinase	*CASK*	8573	1.34
caveolin 1	*CAV1*	857	-1.80
coiled-coil and C2 domain containing 1A	*CC2D1A*	54862	1.27

cellular communication network factor 2	*CCN2*	1490	-1.45
CD40 molecule	*CD40*	958	-3.86
CD47 molecule	*CD47*	961	-1.38
CD99 molecule (Xg blood group)	*CD99*	4267	2.02
cyclin dependent kinase 1	*CDK1*	983	-1.43
cyclin dependent kinase inhibitor 2A	*CDKN2A*	1029	-2.17
checkpoint kinase 1	*CHEK1*	1111	-1.31
cytokine induced apoptosis inhibitor 1	*CIAPIN1*	57019	-1.47
collagen type XVII alpha 1 chain	*COL17A1*	1308	2.23
collagen type III alpha 1 chain	*COL3A1*	1281	5.46
collagen type IV alpha 1 chain	*COL4A1*	1282	-1.42
collagen type IV alpha 2 chain	*COL4A2*	1284	-1.37
cytoplasmic polyadenylation element binding protein 2	*CPEB2*	132864	3.89
C-X-C motif chemokine receptor 4	*CXCR4*	7852	1.72
DAB adaptor protein 2	*DAB2*	1601	1.56
death associated protein 3	*DAP3*	7818	-1.63
DNA damage inducible transcript 3	*DDIT3*	1649	-1.75
dicer 1, ribonuclease III	*DICER1*	23405	-1.39
ephrin A3	*EFNA3*	1944	1.72
ephrin B1	*EFNB1*	1947	1.94
early growth response 1	*EGR1*	1958	-1.77
engrailed homeobox 1	*EN1*	2019	2.31
ectonucleotide pyrophosphatase/phosphodiesterase 2	*ENPP2*	5168	2.40
F2R like trypsin receptor 1	*F2RL1*	2150	1.44
Fas cell surface death receptor	*FAS*	355	-1.47
fibulin 5	*FBLN5*	10516	1.72
FERM domain containing kindlin 2	*FERMT2*	10979	-1.65
fibroblast growth factor 5	*FGF5*	2250	2.67
fibronectin 1	*FN1*	2335	1.76
FUS RNA binding protein	*FUS*	2521	1.36
GATA binding protein 3	*GATA3*	2625	1.58
growth differentiation factor 15	*GDF15*	9518	2.89
GDNF family receptor alpha 1	*GFRA1*	2674	3.18
GDNF family receptor alpha 2	*GFRA2*	2675	5.71
glutathione peroxidase 1	*GPX1*	2876	-1.34

glutamate metabotropic receptor 8	*GRM8*	2918	74.05
heparin binding EGF like growth factor	*HBEGF*	1839	1.81
heparin binding growth factor	*HDGF*	3068	-1.56
homocysteine inducible ER protein with ubiquitin like domain 1	*HERPUD1*	9709	-2.11
hepatocyte growth factor	*HGF*	3082	39.27
major histocompatibility complex, class I, A	*HLA-A*	3105	8.78
high mobility group box 2	*HMGB2*	3148	1.56
heme oxygenase 2	*HMOX2*	3163	1.50
homeobox A7	*HOXA7*	3204	2.28
HRas proto-oncogene, GTPase	*HRAS*	3265	1.87
hydroxysteroid 17-beta dehydrogenase 10	*HSD17B10*	3028	-1.80
HtrA serine peptidase 1	*HTRA1*	5654	-3.54
insulin degrading enzyme	*IDE*	3416	-1.30
insulin like growth factor 2	*IGF2*	3481	13.34
insulin like growth factor binding protein 5	*IGFBP5*	3488	2.06
interleukin 11	*IL11*	3589	2.92
interleukin 11 receptor subunit alpha	*IL11RA*	3590	-1.60
interleukin 6 receptor	*IL6R*	3570	-1.96
integrin linked kinase	*ILK*	3611	1.34
integrin subunit alpha 1	*ITGA1*	3672	-1.64
integrin subunit alpha 4	*ITGA4*	3676	-1.48
integrin subunit alpha 8	*ITGA8*	8516	2.23
integrin subunit alpha V	*ITGAV*	3685	-1.39
integrin subunit beta 1	*ITGB1*	3688	-1.52
jagged canonical Notch ligand 1	*JAG1*	182	1.53
junctional adhesion molecule 3	*JAM3*	83700	-1.42
Jun proto-oncogene, AP-1 transcription factor subunit	*JUN*	3725	1.27
L1 cell adhesion molecule	*L1CAM*	3897	1.94
LIF interleukin 6 family cytokine	*LIF*	3976	1.48
leucine rich repeat and Ig domain containing 1	*LINGO1*	84894	1.56
lamin A/C	*LMNA*	4000	-1.35
LIM homeobox transcription factor 1 beta	*LMX1B*	4010	1.52
mesencephalic astrocyte derived neurotrophic factor	*MANF*	7873	-1.45
mitogen-activated protein kinase kinase kinase 12	*MAP3K12*	7786	1.81
microtubule associated protein tau	*MAPT*	4137	1.79

MET proto-oncogene, receptor tyrosine kinase	*MET*	4233	-1.37
muskelin 1	*MKLN1*	4289	-1.26
mucin 4, cell surface associated	*MUC4*	4585	2.75
MYB binding protein 1a	*MYBBP1A*	10514	-1.31
MYCN proto-oncogene, bHLH transcription factor	*MYCN*	4613	4.83
nuclear receptor coactivator 3	*NCOA3*	8202	-1.30
neurofilament light chain	*NEFL*	4747	4.50
nestin	*NES*	10763	1.47
neurite extension and migration factor	*NEXMIF*	340533	4.54
nerve growth factor	*NGF*	4803	3.89
nerve growth factor receptor	*NGFR*	4804	2.11
nucleolar protein 3	*NOL3*	8996	-2.05
nephronectin	*NPNT*	255743	-1.45
neurotrophic receptor tyrosine kinase 2	*NTRK2*	4915	3.34
opsin 3	*OPN3*	23596	-2.53
parvin gamma	*PARVG*	64098	19.50
protocadherin gamma subfamily A, 4	*PCDHGA4*	56111	3.29
protocadherin gamma subfamily B, 1	*PCDHGB1*	56104	2.04
phosphodiesterase 6B	*PDE6B*	5158	11.27
proenkephalin	*PENK*	5179	-18.47
phosphatidylinositol-4,5-bisphosphate 3-kinase catalytic subunit alpha	*PIK3CA*	5290	-1.39
phospholipase A2 group IVA	*PLA2G4A*	5321	5.32
PLAG1 like zinc finger 1	*PLAGL1*	5325	-1.38
plasminogen activator, urokinase	*PLAU*	5328	-1.79
plasminogen activator, urokinase receptor	*PLAUR*	5329	1.56
plectin	*PLEC*	5339	1.28
PTPRF interacting protein alpha 2	*PPFIA2*	8499	3.97
protein kinase cAMP-activated catalytic subunit alpha	*PRKACA*	5566	1.31
patched 1	*PTCH1*	5727	2.62
phosphatase and tensin homolog	*PTEN*	5728	-1.79
prostaglandin-endoperoxide synthase 2	*PTGS2*	5743	7.18
pleiotrophin	*PTN*	5764	22.80
radixin	*RDX*	5962	-1.68
REL proto-oncogene, NF-kB subunit	*REL*	5966	-1.41
ret proto-oncogene	*RET*	5979	4.26

ras homolog family member B	*RHOB*	388	2.81
sterile alpha and TIR motif containing 1	*SARM1*	23098	1.79
semaphorin 3E	*SEMA3E*	9723	-1.86
serpin family I member 1	*SERPINI1*	5274	-2.53
SHC adaptor protein 1	*SHC1*	6464	-1.55
SHC adaptor protein 3	*SHC3*	53358	4.94
sirtuin 3	*SIRT3*	23410	1.55
sirtuin 5	*SIRT5*	23408	-1.55
solute carrier family 1 member 2	*SLC1A2*	6506	3.37
solute carrier family 1 member 3	*SLC1A3*	6507	-1.35
solute carrier family 8 member A3	*SLC8A3*	6547	11.18
snail family transcriptional repressor 2	*SNAI2*	6591	8.04
synaptosome associated protein 25	*SNAP25*	6616	2.94
superoxide dismutase 1	*SOD1*	6647	1.35
syntaxin 1B	*STX1B*	112755	7.49
syntaxin binding protein 1	*STXBP1*	6812	1.45
TEK receptor tyrosine kinase	*TEK*	7010	13.63
transforming growth factor beta 1	*TGFB1*	7040	2.31
transforming growth factor beta 2	*TGFB2*	7042	1.70
transglutaminase 2	*TGM2*	7052	-2.47
TIAM Rac1 associated GEF 1	*TIAM1*	7074	1.44
translocase of inner mitochondrial membrane 10B	*TIMM10B*	26515	1.72
TIMP metallopeptidase inhibitor 1	*TIMP1*	7076	-1.26
talin 1	*TLN1*	7094	-1.32
transmembrane protein 106B	*TMEM106B*	54664	-1.63
tenascin C	*TNC*	3371	3.49
TNF receptor superfamily member 10b	*TNFRSF10B*	8795	1.67
tribbles pseudokinase 3	*TRIB3*	57761	-1.50
TSC complex subunit 1	*TSC1*	7248	-1.69
ubiquilin 2	*UBQLN2*	29978	1.60
vinculin	*VCL*	7414	-1.42
tyrosine 3-monooxygenase/tryptophan 5-monooxygenase activation protein zeta	*YWHAZ*	7534	-1.68
zinc finger protein 367	*ZNF367*	195828	1.31
zyxin	*ZYX*	7791	1.44

Supplementary Table 3: Profiles of the proteins in the Ingenuity Pathway Analysis-based proteomic network

Protein name	Protein symbol	Gene symbol	Uniprot ID	Fold change
activity dependent neuroprotector homeobox	ADNP	*ADNP*	Q9H2P0	1.31
AKT serine/threonine kinase 1	AKT1	*AKT1*	P31749	1.29
annexin A2	ANXA2	*ANXA2*	P07355	-1.21
annexin A7	ANXA7	*ANXA7*	P20073	-1.31
BCL2 like 11	B2L11	*BCL2L11*	O43521	-1.21
baculoviral IAP repeat containing 5	BIRC5	*BIRC5*	O15392	-1.43
calreticulin	CALR	*CALR*	P27797	-1.43
calcium/calmodulin dependent serine protein kinase	CSKP	*CASK*	O14936	1.33
caveolin 1	CAV1	*CAV1*	Q03135	-1.51
coiled-coil and C2 domain containing 1A	C2D1A	*CC2D1A*	Q6P1N0	1.21
cellular communication network factor 2	CCN2	*CCN2*	P29279	-2.07
CD47 molecule	CD47	*CD47*	Q08722	-1.78
cyclin dependent kinase 2	CDK2	*CDK2*	P24941	1.43
cyclin dependent kinase inhibitor 2A	ARF	*CDKN2A*	P42771	-1.31
checkpoint kinase 2	CHK2	*CHEK2*	O96017	1.24
cytokine induced apoptosis inhibitor 1	CPIN1	*CIAPIN1*	Q6FI81	-1.92
CRK like proto-oncogene, adaptor protein	CRKL	*CRKL*	P46109	-1.32
cathepsin D	CATD	*CTSD*	P07339	1.56
cortactin	SRC8	*CTTN*	Q14247	-1.64
discs large MAGUK scaffold protein 1	DLG1	*DLG1*	Q12959	-1.29
dynamin 1 like	DNM1L	*DNM1L*	O00429	2.11
ephrin B1	EFNB1	*EFNB1*	P98172	-1.24
epidermal growth factor receptor	EGFR	*EGFR*	P00533	-1.36
erb-b2 receptor tyrosine kinase 2	ERBB2	*ERBB2*	P04626	1.23
Fas associated via death domain	FADD	*FADD*	Q13158	-1.58
Fas cell surface death receptor	TNR6	*FAS*	P25445	-1.66
fibrinogen beta chain	FIBB	*FGB*	P02675	1.38
heparin binding growth factor	HDGF	*HDGF*	P51858	-1.58
hexokinase 2	HXK2	*HK2*	P52789	1.39
HRas proto-oncogene, GTPase	RASH	*HRAS*	P01112	1.33
hydroxysteroid 17-beta dehydrogenase 10	HCD2	*HSD17B10*	Q99714	-1.92

heat shock protein family B (small) member 1	HSPB1	*HSPB1*	P04792	-1.96
HtrA serine peptidase 2	HTRA2	*HTRA2*	O43464	-1.26
huntingtin	HD	*HTT*	P42858	1.43
inhibitor of nuclear factor kappa B kinase regulatory subunit gamma	NEMO	*IKBKG*	Q9Y6K9	-1.48
integrin linked kinase	ILK	*ILK*	Q13418	1.53
integrin subunit alpha 1	ITA1	*ITGA1*	P56199	-1.49
integrin subunit alpha V	ITAV	*ITGAV*	P06756	-1.57
integrin subunit beta 1	ITB1	*ITGB1*	P05556	-1.59
junctional adhesion molecule 3	JAM3	*JAM3*	Q9BX67	-1.83
kinesin family member 2A	KIF2A	*KIF2A*	O00139	1.61
LIM zinc finger domain containing 1	LIMS1	*LIMS1*	P48059	1.86
lamin A/C	LMNA	*LMNA*	P02545	-1.69
mesencephalic astrocyte derived neurotrophic factor	MANF	*MANF*	P55145	-1.85
mitogen-activated protein kinase kinase kinase kinase 4	M4K4	*MAP4K4*	O95819	1.57
mitogen-activated protein kinase 1	MK01	*MAPK1*	P28482	1.29
mitogen-activated protein kinase 9	MK09	*MAPK9*	P45984	1.27
microtubule associated protein tau	TAU	*MAPT*	P10636	1.42
midkine	MK	*MDK*	P21741	1.40
misshapen like kinase 1	MINK1	*MINK1*	Q8N4C8	1.48
mechanistic target of rapamycin kinase	MTOR	*MTOR*	P42345	1.20
N-myc downstream regulated 1	NDRG1	*NDRG1*	Q92597	-1.97
neurofilament light chain	NFL	*NEFL*	P07196	2.70
neurofibromin 1	NF1	*NF1*	P21359	1.28
nidogen 1	NID1	*NID1*	P14543	-1.39
Parkinsonism associated deglycase	PARK7	*PARK7*	Q99497	-1.64
protein disulfide isomerase family A member 3	PDIA3	*PDIA3*	P30101	-2.04
prohibitin 2	PHB2	*PHB2*	Q99623	1.27
plectin	PLEC	*PLEC*	Q15149	1.26
PML nuclear body scaffold	PML	*PML*	P29590	-1.25
PTPRF interacting protein alpha 1	LIPA1	*PPFIA1*	Q13136	1.32
peptidylprolyl isomerase D	PPID	*PPID*	Q08752	-1.41
protein kinase cAMP-activated catalytic subunit alpha	KAPCA	*PRKACA*	P17612	1.76
protein kinase C alpha	KPCA	*PRKCA*	P17252	1.30
protein kinase C delta	KPCD	*PRKCD*	Q05655	1.50
prion protein (Kanno blood group)	PRIO	*PRNP*	P04156	-1.41

proteasome activator subunit 3	PSME3	*PSME3*	P61289	-1.44
protein tyrosine kinase 2	FAK1	*PTK2*	Q05397	1.28
protein tyrosine phosphatase non-receptor type 6	PTN6	*PTPN6*	P29350	1.28
RAS p21 protein activator 1	RASA1	*RASA1*	P20936	1.47
RB transcriptional corepressor 1	RB	*RB1*	P06400	1.29
radixin	RADI	*RDX*	P35241	-1.30
ras homolog family member A	RHOA	*RHOA*	P61586	2.50
ras homolog family member B	RHOB	*RHOB*	P62745	1.85
sirtuin 5	SIR5	*SIRT5*	Q9NXA8	-1.38
synuclein alpha	SYUA	*SNCA*	P37840	-1.37
superoxide dismutase 1	SODC	*SOD1*	P00441	1.37
sorbin and SH3 domain containing 1	SRBS1	*SORBS1*	Q9BX66	-1.27
SRC proto-oncogene, non-receptor tyrosine kinase	SRC	*SRC*	P12931	1.26
syntaxin binding protein 1	STXB1	*STXBP1*	P61764	1.71
TAR DNA binding protein	TADBP	*TARDBP*	Q13148	1.25
transcription factor Dp-1	TFDP1	*TFDP1*	Q14186	-1.31
translocase of inner mitochondrial membrane 10B	T10B	*TIMM10B*	Q9Y5J6	1.22
talin 1	TLN1	*TLN1*	Q9Y490	-1.28
transmembrane protein 106B	T106B	*TMEM106B*	Q9NUM4	-1.48
TNF receptor superfamily member 10b	TR10B	*TNFRSF10B*	O14763	1.26
tumor protein p53	P53	*TP53*	P04637	-1.67
tumor protein p73	P73	*TP73*	O15350	1.26
tropomyosin 1	TPM1	*TPM1*	P09493	-2.09
TSC complex subunit 2	TSC2	*TSC2*	P49815	1.39
ubiquilin 2	UBQL2	*UBQLN2*	Q9UHD9	-1.27
UPF1 RNA helicase and ATPase	RENT1	*UPF1*	Q92900	1.23
vinculin	VINC	*VCL*	P18206	-1.70
tyrosine 3-monooxygenase/tryptophan 5-monooxygenase activation protein zeta	1433Z	*YWHAZ*	P63104	-1.27
zinc finger E-box binding homeobox 1	ZEB1	*ZEB1*	P37275	-1.72
